# Nonlinear Optics in Microspherical Resonators

**DOI:** 10.3390/mi11030303

**Published:** 2020-03-13

**Authors:** Gabriele Frigenti, Daniele Farnesi, Gualtiero Nunzi Conti, Silvia Soria

**Affiliations:** 1 Centro Fermi—Museo Storico della Fisica e Centro Studi e Ricerche “Enrico Fermi”, Compendio del Viminale, Piazza del Viminale 1, 00184 Roma, Italy; g.frigenti@ifac.cnr.it (G.F.); g.nunziconti@ifac.cnr.it (G.N.C.); 2 CNR-IFAC, Istituto di Fisica Applicata “Nello Carrara”, Consiglio Nazionale delle Ricerche, via Madonna del Piano 10, I50019 Sesto Fiorentino (FI), Italy; d.farnesi@ifac.cnr.it; 3 Laboratorio Europeo di Spettroscopia Nonlineare (LENS) - Università degli Studi di Firenze, via Nello Carrara 1, I50019 Sesto Fiorentino (FI), Italy

**Keywords:** kerr nonlinearity, whispering gallery mode, optical resonators, stimulated brillouin scattering, optomechanical oscillations

## Abstract

Nonlinear frequency generation requires high intensity density which is usually achieved with pulsed laser sources, anomalous dispersion, high nonlinear coefficients or long interaction lengths. Whispering gallery mode microresonators (WGMRs) are photonic devices that enhance nonlinear interactions and can be exploited for continuous wave (CW) nonlinear frequency conversion, due to their capability of confine light for long time periods in a very small volume, even though in the normal dispersion regime. All signals must be resonant with the cavity. Here, we present a review of nonlinear optical processes in glass microspherical cavities, hollow and solid.

## 1. Introduction

Optical resonators have been gaining a lot of interest in recent decades in all branches of modern optics, both linear and nonlinear optics [1]. Among these resonators whispering gallery modes resonators (WGMR) have shown high mode stability and high quality factors, up to $10^{11}$. Their fabrication is rather simple and inexpensive. WGMR are total internal reflection resonators and they were first introduced by Lord Rayleigh for sound waves propagating close to the dome wall in St. Paul’s cathedral, London [2]. The same phenomenology can be applied to the optical domain, and it was analyzed in depth by Mie and Debye. The geometry of the resonator determines the volume and field distribution of the modes. This kind of monolithic resonators are excellent platforms for fundamental and applied studies of nonlinear interactions between light and matter mainly due to their long photon lifetimes (long temporal confinement) and their small mode volumes (spatial confinement). Temporal and spatial confinement have made possible optical frequency conversion with low-power continuous wave (CW) lasers with powers ranging from micro-watts to milliwatts. However, the high circulating intensities inside a WGMR are not a sufficient condition for efficient harmonic generation, parametric and hyper-parametric oscillations: these phenomena require fulfilling phase and mode matching and energy conservation conditions [3,4].

**Figure 1:**
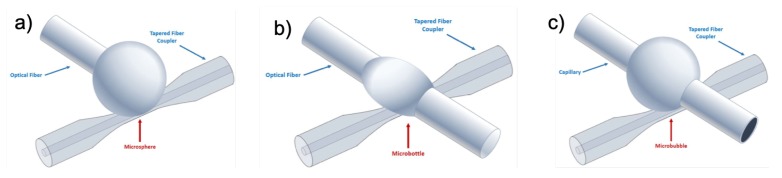
Schematic drawing of microspherical WGMR: (**a**) Microsphere, (**b**) Microbottle and (**c**) Microbubble. The coupling tapered fiber is also sketched in this picture.

This review will describe the applications in nonlinear optics of silica microspherical WGMR, both solid and hollow microcavities. Figure 1 shows an illustration of the three types of WGMR reviewed in this paper. These 3D WGMR show very high quality factor *Q*, their fabrication is easy and regarding specifically nonlinear frequency generation, their very dense mode spectra eases the phase-matching processes required for parametric and hyper-parametric interactions. Microspheres are the first monolithic WGMR that were investigated theoretically and can be obtained by melting amorphous materials such as silica [5]. Hollow microspherical WGMR or the so-called microbubbles (MBR) [6] are also an important family of WGMR which can be tuned beyond a free spectral range (FSR) by changing their radius, either via piezo-electric [7] or gas pression stress [8,9]. Tuning the FSR and the generated combs can be very important for practical applications such as gas sensing or molecular spectroscopy. However, the size of a solid silica microsphere or microbottles is very difficult to change once it is fabricated. A successful way to tune solid WGMR is by coating them with functional materials, such as nanoparticles [10,11]. Hybrid microspheres were also used for Kerr switching [12,13].

Silica glass is a centrosymmetric material; therefore, second order nonlinear interactions are forbidden. Here, the elemental nonlinear interaction is due to the third-order susceptibility $\chi^{3}$ effects, in which four photons are coupled. Previous work in this area, however, has been focused on toroidal WGMRs, where most of the excitable modes are constrained to be the equatorial ones [14], or on highly nonlinear materials [3,15]. Efficient generation of visible light via third-order sum-frequency generation (TSFG) or four-wave mixing (FWM), and third-harmonic generation (THG) in silica microspherical WGMR have been explored. MBR were also studied in the THz domain [16] or for generating two-photon fluorescence (TPF) of the filling liquid [17]. Detailed description of their properties can be found in several books and reviews [1,18,19].

**Figure 2:**
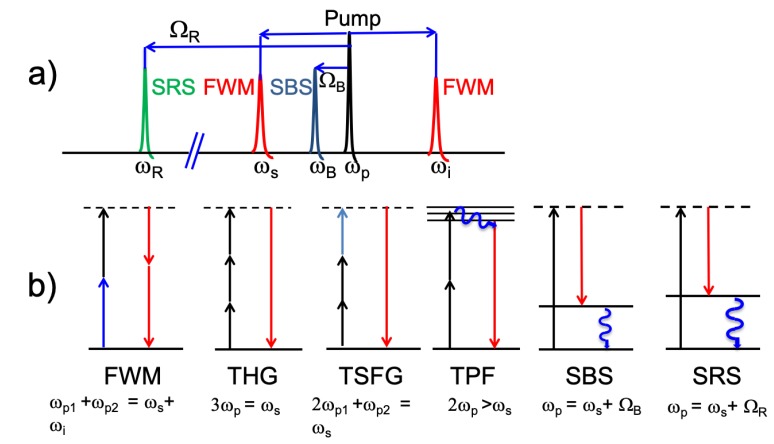
(**a**) Illustration of the infrared spectra observed when nonlinear phenomena are generated. (**b**) Schematic representation of the energy levels of different nonlinear processes (from left to right): FWM, THG, TSFG, TPF, SBS and SRS. Real energy levels are denoted by solid lines (non-parametric processes) while virtual states are indicated by dashed lines.

In this review, several nonlinear effects in silica microspheres will be illustrated (see Figure 2). Generally, nonlinear processes are classified into parametric (hyper-parametric) and non-parametric processes. Parametrical processes are predominant for non or near resonant interactions where the initial and final quantum states are the same, which means that there is no real absorption of photons by the material. Since these processes involve only virtual energy levels, their lifetimes are extremely short (less than a femtosecond). On the other hand, non-parametric processes involve real energy levels with different initial and final quantum states. In this case, there is energy transfer from the photons to the host medium with a relative longer lifetime and it is predominant for resonant interactions. Harmonic generation, TSFG, FWM and coherent anti-Stokes Raman spectroscopy (CARS) are parametric interactions whereas stimulated Raman scattering (SRS) and stimulated Brilluoin scattering (SBS) are non-parametric processes (see Figure 2b).

We will start from tunable optical harmonic generation with extremely narrow linewidth. In the most general case of TSFG, three different waves interact with a nonlinear medium to generate a fourth wave of different frequency ($\omega_{TSFG}=\omega_{1}+\omega_{2}+\omega_{3}$). If the three input frequencies are degenerate, the result will be the THG; in this case the energy conservation requires $\omega_{THG}=3\omega_{1}$. The additional phase-matching condition requires $n\left(\omega_{THG}\right)=n\left(\omega_{p}\right)$, which in general can be fulfilled, because linear and nonlinear dispersion can be compensated for by the dense distribution of degenerate whispering gallery modes (WGMs) with different polar number and decreasing effective index $n_{eff}=m/kR$ [1]. In a WGMR, an additional boundary condition leads to a strict value for the resonant frequency, which may be in conflict with the strict energy conservation, meaning that $\omega_{TSFG}$ or $\omega_{THG}$ is out of resonance.

Stimulated Raman scattering (SRS) is a pure gain process and, therefore, the phase-matching condition is established automatically. Stimulated anti-Stokes Raman scattering (SARS) has been observed in single-component microdroplets with strong Raman gain [20] and from minority species in multi-component microdroplets with low Raman gain [21]; in the latter case, external seeding at the Stokes frequency was used to enhance the SARS signal. However, SARS does require phase-matching, to be efficiently generated and it is thresholdless. WGMRs provide ultra-low thresholds [22] for SRS generation which is a huge advantage. In consequence, one can find extensive literature about SRS generation in WGMRs, but not SARS. SARS and CARS are four-wave mixing (FWM) processes, where vibrational transition frequencies match the beating frequencies between the involved waves, usually, the pump and the anti-Stokes (or Stokes) waves. Cavity resonant enhanced SARS generation, multi-order SARS, and third-order nonlinear processes in silica WGMRs at any dispersion value [23,24] were presented by Farnesi et al. [25]. The linewidth of the cavity-enhanced SARS emission is similar to that of SRS and the pump, and it is a high-quality mode [26]. SBS is an inelastic scattering process like SRS but it is a coherent interaction of light photons and acoustic phonons instead of light photons and light phonons (SRS). The photon-phonon interaction is enhanced due to the overlap of both waves inside the WGMR, which acts as a dual photonic-phononic cavity, named also as phoXonic cavity [27,28]. Another important difference between SBS and SRS are the gain bandwidth and gain coefficient. SBS has one of the largest gain coefficients but a narrower gain bandwidth compared to SRS that constrains WGMR geometries, since the Brillouin frequency shift should match the free spectral range (FSR) of the resonator. It has been demonstrated that this constrain can be bypassed by using higher-order modes [18,29]. All these nonlinear phenomena can occur simultaneously despite their different lifetimes. It has been observed in conjunction THG, SRS and TSFG; or SBS and FWM.

## 2. Kerr Effects

Silica glass is a centrosymmetric material with an odd-order nonlinear polarization which is directly proportional to the nonlinear third-order susceptibility $\chi_{\left(3\right)}$ of the hosting material. The $\chi_{\left(3\right)}$ is a fourth order tensor with 81 elements. The third-order nonlinear polarization is responsible for several nonlinear phenomena, such as THG, FWM, TSFG, the optical Kerr effect and CARS.

### 2.1. Third-Order Sum-Frequency Generation

The nonlinear polarization for third-order sum-frequency generation (TSFG) [30] can be written as:

(1) $$P^{NLS}\left(r,\omega \right)=\chi^{\left(3\right)}E_{a}\left(r,\omega_{a}\right)E_{b}\left(r,\omega_{b}\right)E_{c}\left(r,\omega_{c}\right)$$

where $E(\omega_{i})$ is the electric field amplitude at frequency $\omega_{i}$. This equation shows that in the third-order approximation, the radiation at the new frequency $\omega =\omega_{a}+\omega_{b}+\omega_{c}$ (energy conservation) can be generated by an intense field containing $\omega_{a}$, $\omega_{b}$, and $\omega_{c}$. This general case, or third-order sum-frequency generation (TSFG) has been studied in WGM structures, in particular in liquid droplets, starting from 1989 [31,32,33]. TSFG is a weak process; even when conditions are optimized, the emission is only $10^{-4}$ times the typical intensity of SRS. A model for TSFG in spherical dielectric microresonators can be based on the work of Chew et al. [34] for emission from a polarization source within a sphere. A particular case of TSFG is the third-harmonic generation (THG) in which the three input frequencies are degenerate and so $\omega_{TGH}=3\omega_{1}$.

The polarization in Equation (1) can be written as

(2) $$P^{NLS}\left(r,\omega \right)=D\sum_{jkl}\chi_{ijkl}^{\left(3\right)}E_{a}\left(r,\omega_{a}\right)E_{b}\left(r,\omega_{b}\right)E_{c}\left(r,\omega_{c}\right)$$

where j,k,l are the three orthogonal coordinate directions and *D* is the number of distinct permutation of $\omega_{a},\omega_{b}$ and $\omega_{c}$. Being silica an isotropic material, only three independent elements can be considered:

(3) $$\chi_{ijkl}=\chi_{1122}\delta_{ij}\delta_{kj}+\chi_{1212}\delta_{ik}\delta_{jl}+\chi_{1212}\delta_{il}\delta_{jk}$$

In spherical coordinates (r,θ,ψ), for the transverse electric field (TE) in a sphere, the radial component is zero and $\chi_{1111}^{\left(3\right)}=\chi_{1122}^{\left(3\right)}+\chi_{1212}^{\left(3\right)}+\chi_{1221}^{\left(3\right)}$.

In the simplest case of THG, $\chi_{1122}^{\left(3\right)}=\chi_{1212}^{\left(3\right)}=\chi_{1221}^{\left(3\right)}$, and so:

(4) $$\chi_{ijkl}^{\left(3\right)}=\chi_{1122}^{\left(3\right)}\left(\delta_{ij}\delta_{kj}+\delta_{ik}\delta_{jl}+\delta_{il}\delta_{jk}\right)$$

and the θ component of the polarization is:

(5) $$P_{\theta }^{NLS}=3\chi_{1122}^{3}\left(E_{a\theta }^{3}+E_{a\theta }E_{a\phi }^{2}\right)$$

The radiations generated by the polarization $P^{NLS}\left(r^{\prime },\omega \right)$, where $r^{\prime }$ is the source position, induce additional fields which have to satisfy the boundary conditions at the surface of the sphere. The solution is a combination of spherical Bessel and Hankel functions, $j_{n}$ and $h_{n}^{1}$, respectively.

If the three waves generating the TSFG are standing waves, then the output is a standing wave too. The fields (TE) can be written as:

(6) $$E_{s}\left(r,\omega_{s}\right)=Ag_{n}j_{n}\left(k_{t}r\right)\left[Y_{nnm}\left(\theta ,\phi \right)+Y_{nnm}^{*}\left(\theta ,\phi \right)\right]/2$$

where *A* is the amplitude factor proportional to $\sqrt{I_{s}}$. The field components are labeled $n_{s}$ and $m_{s}$, where *s* is a,b or *c*.

Obtaining the total power at ω requires the integration over all ω:

(7) $$P_{n_{3}m_{3}}^{T}=\int_{0}^{\infty }P_{n_{3}m_{3}}\left(\omega \right)d\omega$$

**Figure 3:**
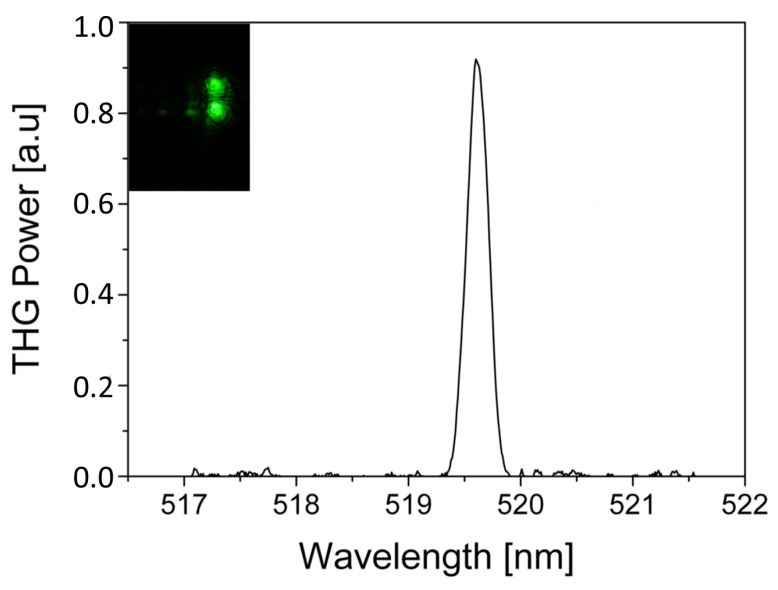
Emission spectrum indicating third-harmonic generation at 519.6 nm when pumping at 1556.9 nm, whereas the inset picture was taken during the spectral measurements. Reproduced with modifications from Ref. [35].

The TSFG power is proportional to the spatial overlap integrals as well as to a frequency overlap integral; the former ones are calculated by integrating the product of the fields of the TSFG mode and of the three generating modes over the sphere volume. In addition to the energy and momentum conservation, in WGMR the pump and the generated frequency must be resonant. When these three conditions are fulfilled, the high quality factor enhances the interaction. The three conditions are quite difficult to be fulfilled simultaneously. The intermodal dispersion of the different spatial WGMs can be used, however, to obtain highly efficient frequency conversion. The inset of Figure 3 shows a picture of the microsphere with the TH signal with the characteristic upper and lower green lobes along the polar direction. As expected, TH signal is codirectional with the pump. Figure 3 shows the emission spectrum of THG at 519.6 nm when pumping with 1556.9 nm.

Asano et al. [36] observed THG in silica microbottle resonators. This particular resonator showed interface and surface effects that allowed the simultaneous generation of THG and second harmonic generation (SHG). In this work, the pump powers are over 200 mW, quite high compared to previous works in other types of WGMR. [25]. A way to lower the launched pump power into the microresonator is coating its surface. Dominguez et al. [37] used a similar strategy for second harmonic generation, coating the silica microspheres with a crystal violet monolayer. Chen et al. [38] have published very recently impressive results in terms of efficiency. The authors have coated silica microspheres with a thin layer of 4-[4-diethylamino(styril)]pyridium (DSP) molecules. DSP has a high third-order nonlinear coefficient and the efficiency of THG is 4 order of magnitude higher than the reported in bare silica microspheres. The authors also observed multiemissions due to TSFG.

### 2.2. Four-Wave Mixing

Third-order four-wave mixing (FWM) is a hyper-parametric oscillation where two pump photons $\omega_{pump}$ generate a signal $\omega_{S}$ and an idler photon $\omega_{I}$. It requires two conditions to be satisfied: the momentum conservation, which is intrinsically satisfied in WGM resonators [39], and the energy conservation, which is not a priori satisfied since the separation between adjacent modes $\nu_{FSR}=\left|\nu_{m}-\nu_{m+1}\right|$ can vary due to the material and cavity dispersion. Indeed, only in recent works this process has been observed by coupling a CW laser into microcavities exploiting the Kerr nonlinearity to enable cascaded four-wave mixing. The resonances of the WGMR will also impose that the new generated frequencies will be discrete, creating a frequency comb.

The comb generation can occur in two different ways: as a Type I (or natively mode spaced comb), with sidebands separated by one free spectral range (FSR), and a Type II (or multimode spaced comb), with sidebands separated from the pump by several FSRs. Cascaded FWM preserves the initial spacing to higher-order emerging sidebands thank to the conservation of the energy in the parametric processes [40].

The Kerr comb formation starts by generation of the first symmetrical lines generated in a degenerate FWM process when the parametric gain overcomes the loss of the cavity. The separation of the new lines from the pump depends on the dispersion and the pump power. The threshold of the parametric frequency conversion [24], at which the gain of the excited sidebands is equal to the cavity decay rate, is:

(8) $$P_{th}=\frac{\kappa^{2}n_{0}^{2}V_{eff}}{8\eta \omega_{0}cn_{2}}$$

and the gain of the sidebands, for $P_{pump}>P_{th}$, can be written as: [41]

(9) $$G=\sqrt{\kappa^{2}{\left(\frac{P_{abs}}{P_{th}}\right)}^{2}-4{\left(\omega_{0}-\omega_{p}+\mu^{2}D_{2}-\kappa \frac{P_{abs}}{P_{th}}\right)}^{2}}$$

where $P_{abs}$ is the power absorbed by the cavity. At the threshold (G=κ), from Equation (9) we obtain:

(10) $$\sqrt{\frac{f^{2}}{{\left|a_{0}\right|}^{2}-1}}-d_{2}\mu_{th}^{2}+{\left|a_{0}\right|}^{2}-\sqrt{{\left|a_{0}\right|}^{4}-1}=0$$

The theory of generation of frequency combs in silica microspheres has been described in a 2010 article by Chembo et al. [42].

**Figure 4:**
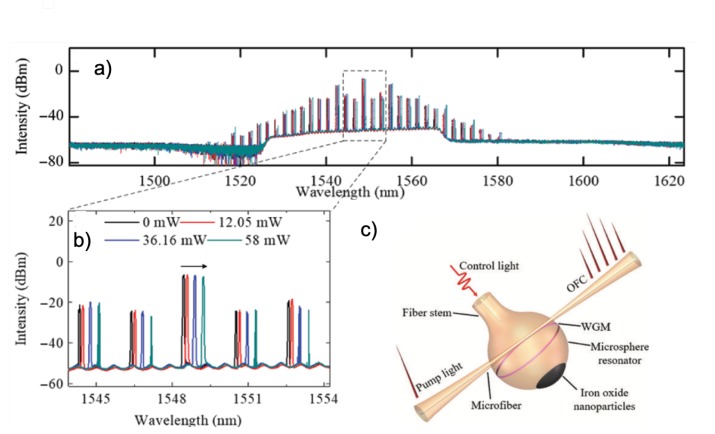
(**a**) Generated Kerr combs in a hybrid microsphere for different control light powers, (**b**) zoom-in of the spectra of panel (**a**,**c**) Sketch of experimental set-up showing an iron oxide nanoparticle coated silica microsphere of 248 μm diameter for photothermal tuning of generated optical frequency combs (OFC). The pink ring is the excited whispering gallery mode and the black polar cap represents the coated area with iron oxide nanoparticles. Reproduced with modifications from Ref. [10].

The first experimental demonstration of FWM in silica microspheres was done by Kippenberg et al. [43]. The authors showed the generation of a pair of signal-idler photons created by two pump photons separated by one FSR with an emission ratio close to unity. Frequency combs in microspheres were first demonstrated by Agha et al. [44] where the first theoretical model was established. The same group published a broader comb in microspheres [45], but their theoretical model only predicted FWM and modulation instabilities in resonators with anomalous dispersion. However, nonlinear hyper-parametrical oscillations have been also achieved in the regime of normal dispersion [23,25,39,46]. This occurrence is due to the cavity boundary conditions that introduce an additional degree of freedom: the frequency detuning of the pump from the eigenmode of the nonlinear resonator [23,47]. Zhang et al. [10] proposed hybrid silica microspheres for generation of tunable Kerr and Raman-Kerr combs. The authors have coated the polar cap of the microsphere with iron oxide nanoparticles. Since the WGM are excited at the equator of the microspheres, far away from the iron oxide nanoparticles, the high quality factor *Q* was not spoiled. The authors fed the control light into the microsphere through the fiber stem (see Figure 4). This control light was absorbed by the iron oxide nanoparticles and due to a strong photothermal effect, the comb was tuned. The achieved tuning of the Kerr comb was about 0.8 nm whereas the Raman-Kerr comb was tuned about 2.67 nm. The proposed photothermal tuning is an all-optical method and has less disadvantages than the mechanical methods [48] which present mechanical interferences and need cryogenic temperatures. However, the tuning range achieved by mechanical methods was about 450 GHz at 10 K for a microsphere of 40 μm diameter.

**Figure 5:**
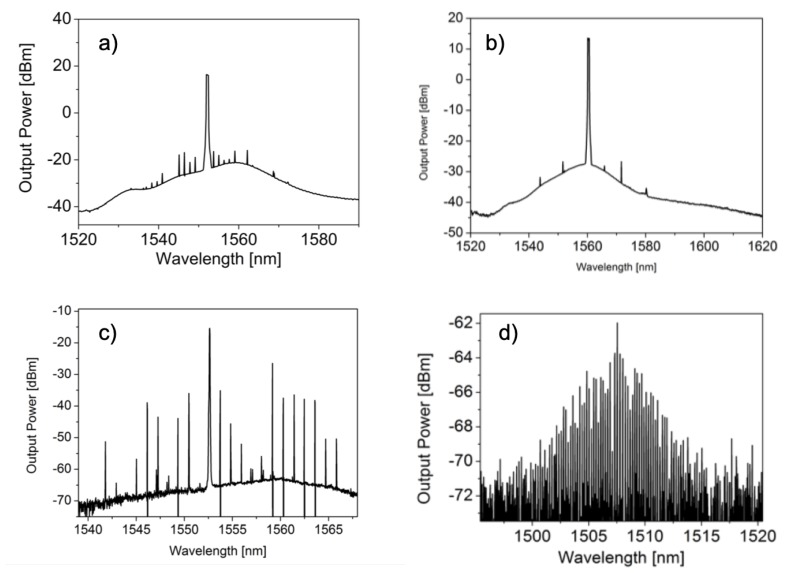
Experimental spectra of: (**a**) a Type I comb, with a frequency offset of 1 FSR, (**b**) a Type II comb, with a frequency offset of 5 FSR, (**c**) FWM in the vicinity of the pump spaced by azimuthal FSR and (**d**) Modulation intensity around the anti-stokes component at 1508 nm, with a frequency offset of 2 vertical FSR (2X0, 12 nm) measured for a microbubble of 475 μm diameter. Reproduced with modifications from Adapted with permission from Ref. [50] © The Optical Society.

For microbubbles, the first demonstration of cascaded FWM was done by Li et al. [49]. Broader combs in microbubbles were demonstrated by Farnesi et al. [50]. The authors here realized a “Type I” or natively mode spaced comb with sidebands separated by one FSR (see Figure 5a) and a “Type II” or multimode spaced comb with sidebands separated by several FSR (see Figure 5b). Figure 5c shows the FWM pairs in the vicinity of the pump at 1.55264 nm in the backward direction. In this case, the FWM pairs are separated by one FSR. At 14 THz from the pump, centered at 1508 nm, an anti-Stokes comb was observed. In this case, the intensity of the anti-stokes component is high enough to generate its own parametrical oscillation, with a separation smaller than the FSR of the cavity. MBRs are spheroidal WGMR with quite dense spectral characteristics with two nearly equidistant mode families characterized by the same azimuthal but different vertical quantum number. The presence of these two mode families gives the different frequency spacing and the asymmetric spectrum (see Figure 5d [50]).

Yang et al. [51,52] have experimentally measured frequency combs in the visible (pump wavelength centered at 765 nm) by engineering the dispersion through wall thickness of the microbubbles and degenerate FWM in hollow microbottles.

### 2.3. Stimulated Raman Scattering

The inelastic scattering of a photon with an optical phonon, which originates from a finite response time of the third-order nonlinear polarization of the material, is called the Raman scattering effect. Spontaneous Raman scattering occurs when a monochromatic light beam propagates in a material like silica. Some of the photons are transferred to new frequencies. The scattered photons may lose (Stokes shift) or gain (anti-Stokes shift) energy.

The left diagram in Figure 2b represents the absorption of a pump photon with energy with the consequent excitation of a molecule from the ground state (*G*) into a higher virtual energy state (*V*). The energy difference between the ground and the excited level is equal to pump photon’s energy. In a second step, the molecule falls to an intermediate level (*I*), which is generated by its own periodical oscillations or rotations. This decay is accompanied by a Stokes photon emission. The destruction of the pump and the generation of the Stokes photon happen simultaneously because *V* is a virtual state. The energy difference between the pump and the Stokes photon is equal to the difference between the energy levels *I* and *G*; the remaining energy is the vibrational energy delivered to the molecule.

In contrast to other kinds of nonlinear phenomena where the molecule returns to its ground level after the interaction, an energy transfer between a photon and a molecule takes place here. Raman scattering is a pure gain process and depends on particular material resonances. In crystalline media, these resonances show a very narrow bandwidth. On the other hand, in amorphous silica the molecular vibration modes are overlapped with each other and create a continuum [53]. Contrary to spontaneous emission, SRS can transform a large part of power into a new frequency-shifted wave with the intensity growing exponentially with the propagation distance in the nonlinear (NL) material. Highly efficient scattering can occur as a result of the stimulated version under excitation by an intense laser beam; 10% or more of the energy of the incident laser beam can be converted.

Generally, in WGM resonators the lasing threshold occurs when the cavity round-trip gain equals round-trip loss. The intra-cavity gain coefficient is related to the bulk Raman gain coefficient $g_{b}$ (in silica the maximum is $6.5\times 10^{14}$ m/W at 1550 nm) through the equation:

(11) $$g_{R}=\frac{c^{2}}{C\left(\Gamma \right)2n^{2}V_{eff}}g_{b}$$

where $V_{eff}$ is the effective modal volume and C(Γ) is the modal coupling. The threshold pump power can be derived by the gain coefficient, taking into account the power build-up factor in the resonator:

(12) $$P_{th}=\frac{\pi^{2}n^{2}V_{eff}}{\lambda_{p}\lambda_{R}C\left(\Gamma \right)g_{R}Q^{2}}$$

Thus, the threshold follows an inverse dependence on the squared quality factor *Q* of the cavity. This explains how an increase in *Q* will cause a two-fold benefit in terms of reducing cavity round-trip losses as well as of increasing the Raman gain, due to the Raman gain dependence on the pump intensity.

Contrary to the parametric effects, Raman scattering is intrinsically phase-matched over the energy levels of the molecule. In other words, it is a pure gain process.

The SRS can be summarized in two parts: (1) the molecular vibrations modulate the refractive index of the medium at the resonant frequency $\omega_{v}$ and the frequency sidebands are induced in the laser field. (2) the Stokes field at frequency $\omega_{S}=\omega_{L}-\omega_{v}$ beats with the laser field to produce the modulation of the total intensity which excites the molecular oscillation at frequency $\omega_{L}-\omega_{S}=\omega_{v}$. In this way the two processes reinforce one another. The Raman emission can be thought as a down-conversion of a pump photon and phonon associated with the vibrational mode of the molecule. The anti-Stokes wave is generated together with the Stokes one, through FWM in which two pump photons annihilate themselves to produce Stokes and anti-Stokes photon can occur if $2\omega_{L}=\omega_{a}+\omega_{s}$, providing the total momentum conservation. This leads to the phase-matching condition $\Delta k=2k\left(\omega_{L}\right)-k\left(\omega_{a}\right)-k\left(\omega_{s}\right)=0$ where k(ω) is the propagation constant. When the phase mismatch Δk is large, Stokes emission experiences gain whereas the anti-Stokes experiences loss. For a perfect phase-matching, the anti-Stokes wave is strongly coupled to the Stokes one, preventing the growth of the latter.

**Figure 6:**
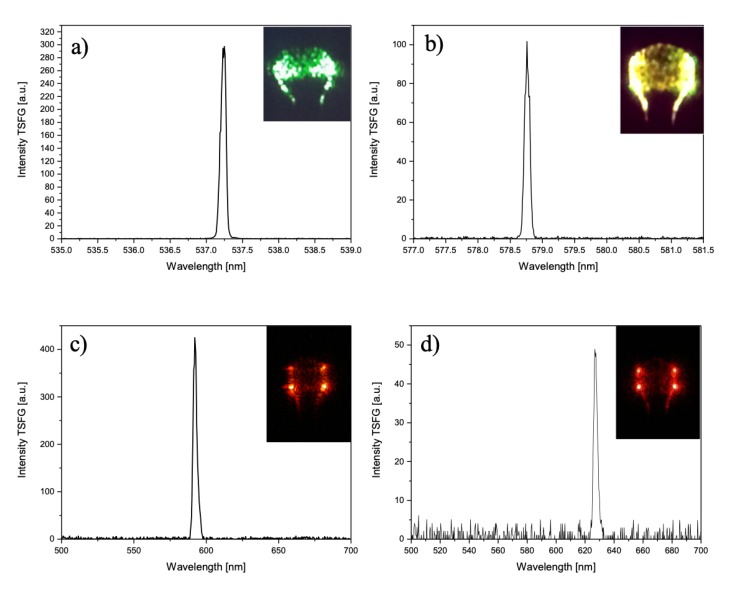
Various spectra obtained with different pump wavelengths showing TSFG standing waves generated among cascaded Raman lines with emission at: (**a**) 537.2 nm; (**b**) 578.8 nm, (**c**) 592 nm, and (**d**) 625 nm. Reproduced with modifications from Ref. [35].

In 2003 a microcavity-based cascaded Raman laser was demonstrated in WGM silica microspheres with sub-milliwatt pump power [54]. In cascaded Raman oscillation, the Raman signals serve to secondary pump field and generate higher-order Raman waves. As the pump power is increased, the first Stokes line extracts power from the pump until it becomes strong enough to seed the generation of a next Stokes line. This process can continue to generate more Raman peaks. The cascade process can be modeled as coupled harmonic oscillators with the pump and the Raman fields by including higher-order coupling terms(see for instance, [55]) The SRS and cascaded SRS in the infrared region occurs as standing waves because the Raman gain that amplifies the waves is the same for waves traveling in either the forward or the backward direction. In the presence of these phenomena, we have also observed TSFG in the visible, obtaining multicolor emission (red, orange, yellow, and green) by tuning the pump wavelength. Figure 6 shows the spectra measured for each different color and the corresponding microscope picture of the microsphere.

**Figure 7:**
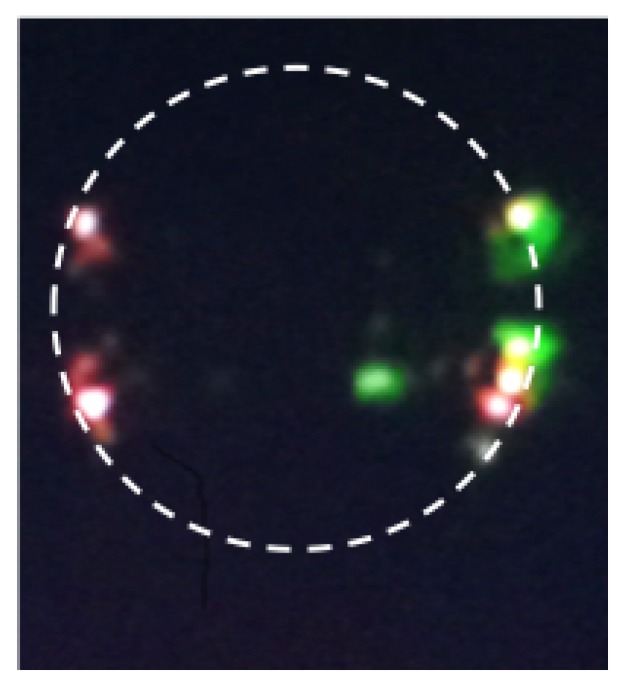
Optical picture of the microsphere showing the TSFG standing wave in the red and the traveling wave in the green of the THG.

In these cases, the pump was high enough to generate several orders of Stokes Raman lines and Raman combs. TSFG and THG can also occur simultaneously. Figure 7 shows the picture of a multicolor emission, one at 519.2 nm and one at 625 nm, corresponding to the THG signal of the pump laser and the TSFG, respectively.

As mentioned before, the red standing wave is the result of TSFG whereas the green traveling wave is TH signal. To fulfill the phase-matching condition and dispersion compensation, we must excite higher-order polar modes (l−∣m∣>1). High polar order modes can be excited by placing the coupling taper far from the equatorial plane where the intensity peaks of these modes are located [1]. These modes also improve mode matching which is another requirement for efficient nonlinear frequency generation. The overlap of the WGM eigenfunctions corresponds to the power of TSFG and THG. In the latter case, the total power is proportional to the overlap of the TH field with the cubic power of the pump field.

**Figure 8:**
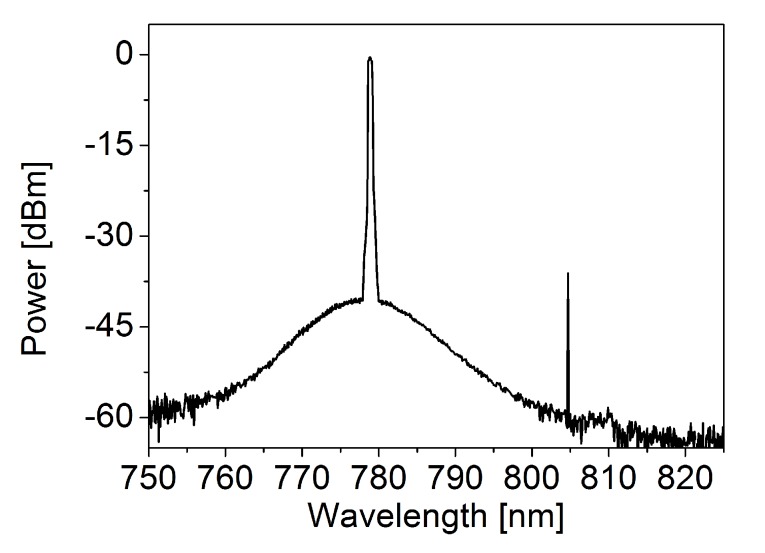
Experimental spectra of cascaded Raman lasing in a microsphere of 50 μm diameter. The laser pump is centered at about 778 nm, the Raman line is centered at about 807 nm.

WGMR Raman lasers can be used in sensing applications. WGMR-based lasers have a very narrow linewidth, they are dopant-free and they can attain high detection resolution down to single nanoparticles [56]. However, biochemical sensors need to work at telecom wavelengths where water shows a very high absorption [57,58]. A way to overcome such limitation is to use laser pumps in the visible or near infrared (NIR) [59,60]. Figure 8 shows the SRS line at 807 nm when a microsphere of about 50 μm diameter is pumped at 778 nm.

Stimulated anti-Stokes Raman scattering (SARS) requires phase-matching, to be efficiently generated, and it is thresholdless. The nonlinear polarization of the anti-Stokes wave, $P_{as}^{NL}$, is defined by the relation:

(13) $$P_{as}^{NL}=\chi^{\left(3\right)}E_{P}^{2}E_{S}^{*}e^{i(2k_{P}-k_{S})z}$$

where $E_{P}$ and $E_{S}$ are the amplitude of the pump and Stokes waves, respectively; $\chi^{3}$ is the third-order nonlinear susceptibility, and $k_{i}$ are the propagation vectors for the pump and Stokes waves [20,21]. Farnesi et al. [25] measured SARS in solid microspherical WGMR in the normal dispersion regime [23,39,46], contrary to well-known theoretical models [44,45], which predicted modulation instabilities and FWM in WGMR only with anomalous dispersion. The cavity boundary conditions introduce an extra degree of freedom, namely the frequency detuning between the pump and the eigenmode of the nonlinear WGMR [23,47]. As stated in [25], there was a negative shift of about 30 MHz of the resonant frequency due to the Kerr effect plus a larger thermo-optic frequency shift. However, the FSR of the resonator showed just a slight change [23]. The linear dispersion also changes slightly since it is calculated as the variation of the FSR [40]. Similar results have been achieved by Soltani et al. [61]. The group have been able to enhance by a factor of 4 the results of Farnesi et al. [25] using hybrid microspheres. In this particular case, silica microspheres have been functionalized with a layer of gold nanorods in polyethilenglycol (PEG). Farnesi et al. [25] have measured some unusual features, namely strong anti-Stokes components and extraordinarily symmetric spectra. Usually in SRS, the Stokes waves are exponentially enhanced, whereas the anti-Stokes waves are exponentially absorbed [62]. Anti-Stokes Raman components are coupled to the Stokes Raman ones [62,63], independently of the magnitude of dispersion. As a result of the coupling and of an effectively phase-matched hyper-parametric process, the anti-Stokes wave grows along the microsphere directly proportioned to the Stokes wave. When there is perfect phase-matching condition, each eigensolution is an equal combination of Stokes and anti-Stokes components with a power ratio of one. When the phase mismatching conditions deviates from zero gradually, we are in an intermediate case where the anti-Stokes/Stokes power ratio is given by the following equation:

(14) $$\frac{P_{a}}{P_{S}}={\left|\frac{\gamma qP}{2\gamma qP+\beta_{2}\Omega^{2}}\right|}^{2}$$

where *P* is the cavity build-up pump power, Ω/2π is the frequency shift between the pump and the first Raman order, $\beta_{2}$ is the linear dispersion, γ is the nonlinearity coefficient and $q=\left(1-\alpha \right)+\alpha \chi^{3}=0.82+i0.25$ is a complex number that depends on the Raman susceptibility of silica and on the fractional contribution of the electronic susceptibility to the total nonlinear index [26,64].

When there is no phase match, the Stokes and anti-Stokes components are effectively decoupled.

**Figure 9:**
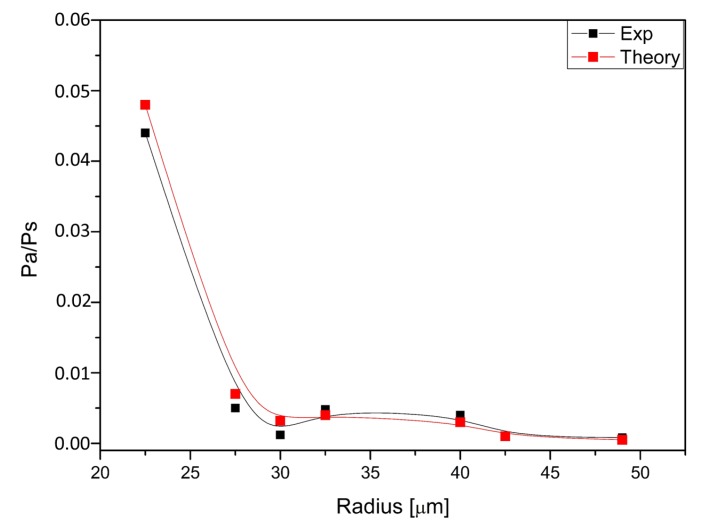
$P_{a}/P_{S}$ ratio: experimental (solid black squares) and calculated values (solid red circles) The lines are a guide to the eye.

This expression is valid for both regimes, normal and anomalous, but the linear dispersion $\Delta k=\beta_{2}\Omega^{2}$ must be large. The values obtained from Equation (14) are in close agreement with the experimental ones, given the uncertainties in $\beta_{2}$ and γ, as it can be seen in Figure 9.

### 2.4. Stimulated Brillouin Scattering

**Figure 10:**
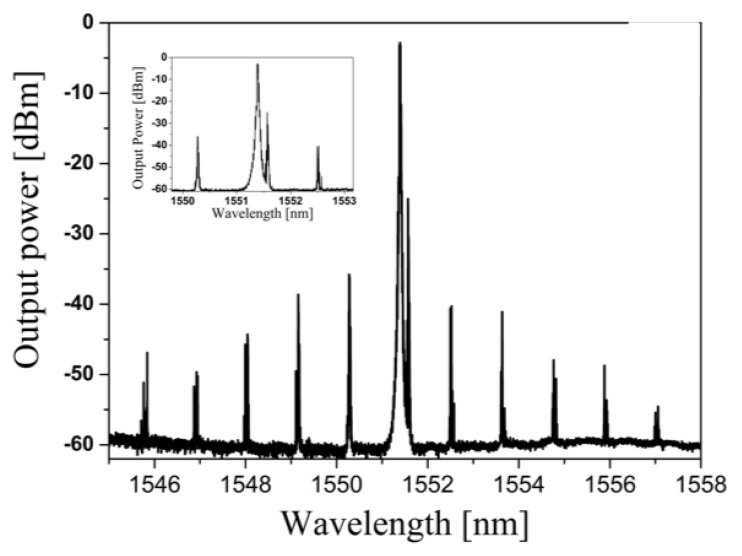
Native Kerr comb and second order SBS in forward direction: pump power 72 mW at 1551,344 nm. (Inset: zoom of the spectrum showing the 2nd order SBS laser line and the first pair of FWM lines) Reproduced with modifications from Ref. [73].

SBS is also an inelastic scattering process but it results from the coherent interaction of light photons and acoustic phonons instead of optical phonons. The WGMR acts as a dual photonic-phononic cavity due to the overlap of both waves inside the resonator. SBS is automatically phase-matched because it is a pure gain process, like SRS. The SBS gain coefficient is one of the largest but with a small gain bandwidth [62]. The narrow bandwidth places very stringent conditions on the geometry of resonators since it would require a Brillouin frequency shift that equals the free spectral range (FSR) of the WGMR. [36,65,66]. We need to excite high order modes [29] with vertical FSR smaller than the fundamental FSR [50] in order to bypass such a stringent condition. SBS can further reduce its threshold power when it is resonantly enhanced, and it can be as low as some micro-watts [65]. The threshold power is directly proportional to the mode volume [67]

(15) $$P_{th}=\frac{\pi^{2}n^{2}V_{eff}}{\lambda_{p}\lambda_{s}Bg_{b}Q_{p}Q_{s}}\frac{1}{1+\frac{Q_{m}\lambda_{m}}{2\pi r}}$$

where the subscripts *p*, *s*, *m* refer to pump, Stokes and mechanical modes, and *B* is the mode overlap. SBS has been demonstrated in silica [67,68] and tellurite [69] microspheres, microbottles [70] and microbubbles [29,71,72]. Hollow and solid spherical WGMR have been used to generate both backward and forward SBS. As with SRS, cascaded SBS can also be generated in WGMR. Even and odd orders are observed in both directions, but showing different lasing efficiency (even (odd) orders are more efficient in forward (backward) direction) [72]. Scattering can occur in forward direction with frequencies in the MHz–GHz range whereas backward are in the GHz range. The SBS frequency in silica glass is about 11 GHz and it scales with the optical one, with a bandwidth in the range of 20–60 MHz at telecom frequencies. In our experiments, the free spectral range (FSR) of our MBR is 141 GHz (diameter about 475 μm) and 105 GHz (diameter about 675 μm). Therefore, we can obtain SBS only by using high order modes, in that case the vertical FSR is much less than the FSR [50]. Frequency combs and SBS can coexist in both forward and backward directions, as Figure 10. SBS efficiency in microbubbles can be so high to allow degenerated FWM from the Brillouin laser line. Figure 10 shows in forward direction, cascaded FWM from the second order Brillouin laser line (1.54522 nm) for a pump wavelength centered at 1.54504 nm

## 3. Kerr Switching in Hybrid Resonators

Inorganic materials still show weak nonlinearity, slow dynamics and the difficulty of discrimination between thermal and Kerr nonlinearity at room temperature limits their performance. Significant advantages can be obtained if organic-inorganic hybrid systems can be used [74,75]. π-conjugated polymers are extremely suitable nonlinear optical materials which show structural flexibility, relative ease of preparation, high $\chi^{3}$ values and high photostability. Hybrid polyfluorene derivatives-silica WGMR have been demonstrated as very good candidates for all-optical switching [12,13] where two beams are present, namely a pump beam that switches a probe [76,77]. The electronic Kerr effect is almost instantaneous (picosecond timescales) and due to the enhancement of the WGMR combined with a strong third-order nonlinearity, the intensities used are well below the damage threshold of the conjugated polymer. The material refractive index *n* and the absorption coefficient α depend on the light intensity *I* in the material according to the equations $n=n_{0}+n_{2}I+n_{4}I^{2}+\ldots$ (with the nonlinear refractive index $n_{2}\approx ℜ\left(\chi^{3}\right)$, $n_{4}\approx ℜ\left(\chi^{5}\right)$, where $\chi^{3}$ and $\chi^{5}$ are the third- and fifth- order nonlinear optical susceptibilities) and $\alpha =\alpha_{0}+\beta I$ (with the nonlinear absorption coefficient $\beta \approx ℑ(\chi^{3})$). All-optical switching for a probe signal $I_{probe}$, which is resonant with the microsphere, can be realized using a resonant pump beam $I_{pump}$, which affects the coated cavity resonance position by changing the refractive index of the coating in the corresponding wavelength range [78].

If the $\chi^{3}$ and $\chi^{5}$ are caused by fast electronic Kerr nonlinearity, then, as mentioned before, the nonlinear switching is on a picosecond time scale, which is the most desired situation for the optical switching. However, thermal nonlinearities can restrict the use of the hybrid devices because the spectral response is sensitive to the input power of the probe signal as well [76]. In that case, the light-induced changes in the refractive index can be described phenomenologically by $n_{2}=\left(\delta n/\delta T\right)T_{L}$, where $T_{L}$ is the laser-induced change of the temperature of the nonlinear medium, the corresponding switching time being about $10^{-3}$–$10^{-5}$ s. In other words, the thermal switching of a nonlinear medium for the case of a standard Ti-sapphire laser should be the same for the pulsed or CW mode operation regime. Moreover, an intrinsically weak but highly localized probe beam can also participate in this type of the light-induced WGM switching.

Murzina et al. [13] reported on the all-optical switching of WGM in silica microspheres with two types of coatings, an active one based on a Kerr polymeric material (polyfluorene derivative, PF(o)n) [12] and an inert polymer based on an anionic copolymer made of methacrylic acid and methyl methacrylate (Eudragit^®^ L100) [79]. The authors modeled the overlap of the coupled optical field with the polymer layer and verified the role of the probe field experimentally for both polymer coatings.

**Figure 11:**
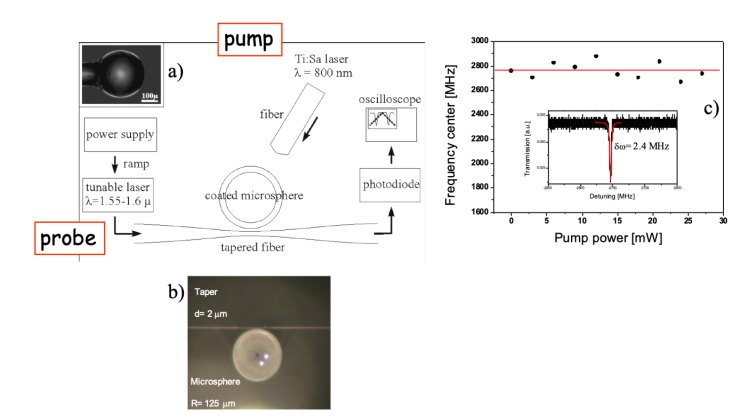
(**a**) Experimental pump-and-probe set-up. Left hand side inset: optical image of the WGMR. (**b**) Picture of the microsphere and the coupling taper; (**c**) Frequency center of the WGMR resonance versus pump power. The red line is a guide to the eye. Inset: Zoom of a typical resonance for a bare microsphere, the red line is a Lorentzian fit with a FWHM of about 2.4 MHz. Reproduced with modifications from [12].

Figure 11 shows an sketch of the set-up, an optical picture of a microspherical WGMR, with a diameter of about 250 μm and the taper; and the control test with a bare WGMR. A Tunics Plus was used as probe beam, which is a semiconductor external-cavity laser tunable in the spectral range of 1.55–1.6 μm and with 300 kHz linewidth. The pump probe was a Mira 900-f Ti-Sapphire (Coherent). The laser probe was coupled into the WGMR through a homemade tapered fiber whereas the laser pump was coupled into a multimode fiber that illuminated a hemisphere of the WGMR. To make a polymer coverage, the dip coating technique was used. The authors obtained layers of about 100 nm thickness for PF(o) coatings and of about 50 nm thickness for Eudragit coatings. The *Q* values were higher than $10^{8}$ for bare microspheres and higher than $10^{6}$ after polymer coating. To attain adequate solubility in common organic solvents and mesogenic behavior, the polyfluorene derivative, PF(o)n, was functionalized at the C9 position of the fluorine ring with two pendant octyl chains [80]. It shows a maximum absorption at $\lambda_{abs}$ = 379 nm, and the measured $n_{6}$ is about $2\times 10^{-10}$ cm${}^{2}$/W and β coefficient $7\times 10^{-7}$ cm/W [8,14]. As the first step, pump-induced effects on the barre WGMR in were studied. No shift was seen as the averaged pump power was increased up to 30 mW. Thus, we may assume that the Kerr nonlinearity and the thermal nonlinear effects of pure fused silica were negligible for these pump-and-probe levels.

**Figure 12:**
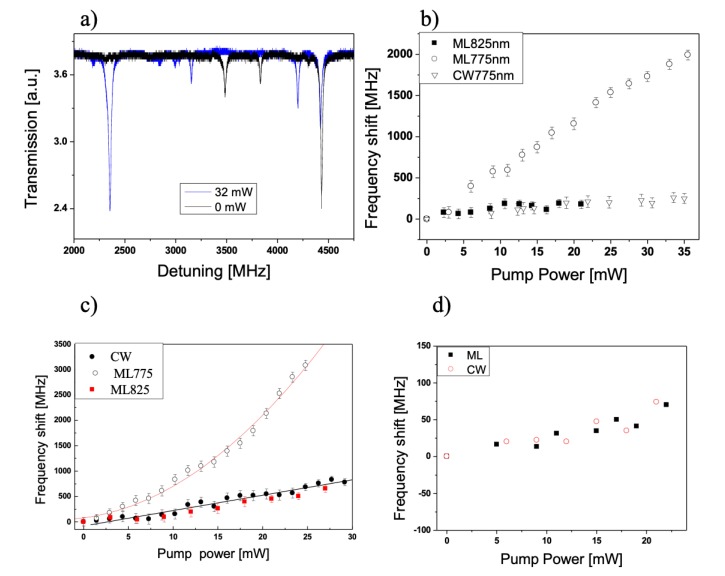
(**a**) Typical WGM spectra measured for a polymer-coated microsphere for two different pump powers: (black) pump laser off and (blue) 32 mW. $\lambda_{pump}$ = 775 nm, $\lambda_{probe}$ = 1600 nm. (**b**) Pump power dependence of the detuning of WGM in PF(o)n coated microspheres for mode-locked regime of the Ti-sapphire pump laser: 825 nm (filled squares) and 775 nm (empty circles); and CW at 775 nm (empty downside triangles). (**c**) The probe wavelength is $\lambda_{probe}$ = 1558 nm, switching for CW (filled circles), mode-locked at 775 nm (empty circles) and mode-locked at 825 nm. (**d**) Eudragit coated microspheres for mode-locked regime of the Ti-sapphire pump laser for two different regimes: CW (empty circles) and pulsed (filled squares). The probe wavelength is $\lambda_{probe}$ = 1558 nm. Reproduced with modifications from [13].

Figure 12a shows the WGM spectrum measured for the probe wavelength of 1600 nm and for two different pump powers for the mode-locked regime of the Ti:Sapphire laser. The Ti:Sapphire was tuned at 775 nm to generate the two-photon absorption (TPA) in the PF(o)n coating of the hybrid WGMR. Thermal nonlinearities can be ruled out since no broadening of the resonance, hysteresis or asymmetries could be observed in the transmission spectra.

The results of the pump-and-probe experiments are shown in Figure 12b, where a frequency shift of 2 GHz is obtained in pulsed regime for an average pump power of 35 mW at 775 nm for a probe of 1600 nm. To discriminate the thermal shift from the Kerr shift, we have performed measurements in CW and pulsed regime for the same average pump powers, similarly to a previous work [12]. For the same wavelength and average pump power, the authors obtained a much lower spectral shift of 250 MHz in the CW regime (Figure 12b). In here, we have also tested the influence of the wavelength of the pump beam. Figure 12b also shows a frequency shift of 200 MHz obtained in the mode-locked regime for an average pump power up to 21 mW at 825 nm. The detuning is of the same order of magnitude as the CW regime. We have chosen 825 nm as a pump beam because two-photon absorption (TPA) is not feasible; and because it is also far from the second harmonic of the probe beam. In that case the pump beam acts as a spectrally broad thermal source only.

Figure 12c shows the frequency shift versus pump power for $\lambda_{probe}$ = 1558 nm at two different laser regimes for the PF(o) coated WGMR. At $\lambda_{pump}$ = 775 nm a clear quadratic dependency can be observed, whereas at $\lambda_{pump}$ = 825 nm the dependency is linear and the detuning is of the same order of magnitude as the CW regime, indicating again that in absence of TPA, the pump acts as a thermal source. It can also be observed that in the case of $\lambda_{probe}$ = 1558 nm the magnitude of the shift is greater than in the case of $\lambda_{probe}$ = 1600 nm, for both regimes, pulsed and CW. Figure 12d shows an almost null red-shift up to 20 mW of pump power for $\lambda_{probe}$ = 1558 nm for a WGMR coated with Eudragit^®^ L100, an inert polymer.

## 4. Two-Photon Fluorescence

Two-Photon Fluorescence (TPF) is a validated technique for imaging and detection of labeled biological material such as peptides [81] and steroids [82,83]. TPF has numerous advantages over conventional one photon fluorescence (OPF), being the most important one the large Stokes shift between emission and excitation. The large energy gap between the fields lowers the background noise. Other advantages are the reduced static photo-bleaching of dyes due to the absorption’s quadratic dependence on the intensity; and the wide range of fluorescent dyes with high quantum yields and molar extinction in the visible. However, TPF requires high photon density flux, reached by tightly focusing the laser light. To avoid tight focusing and achieve the needed intensities, much research has resorted to enhancing photonic platforms such as resonators [84]. MBR are the most suitable WGMR for TPF, being hollow they can be filled with liquids. For the TPF demonstration Pastell et al. [17] filled MBR with a $10^{-3}$ and $10^{-4}$ solution of fluorescein and $10^{-6}$ solution of Rhodamine 6G.

**Figure 13:**
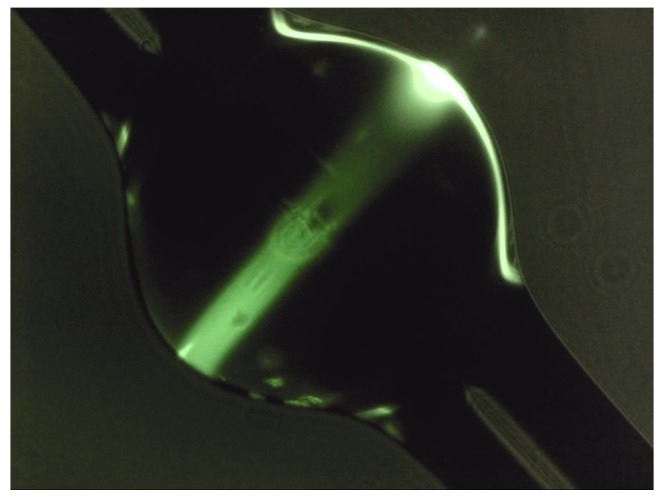
Fluorescence image of the MBR filled with $10^{-3}$ solution of fluorescein, showing the TPF band and lobes, and the fluorescence coupled back into the MBR wall. Reproduced with modifications from [17].

The authors used a modified confocal microscope for coupling the light into the microbubble resonator [85], an inverted light microscope (Nikon Eclipse TE2000U) up-graded to a multi-modal imaging system even though it can be used either as a bright-field microscope or as a phase contrast microscope. The microscope was pumped by a Mira 900-f Ti: Sapphire (repetition rate 76 MHz and 150fs pulse duration, Coherent) with an average power of 1.2 W. It has an average power of 1.2 W and it can be tuned over a wavelength range of 690–950 nm within which falls the two-photon absorption spectra of many fluorophores [86]. The wavelength in the experiments was set to 800 nm. The raster scan of the beam was stopped and it was coupled into the MBR by focusing the laser beam tangential to the bubble wall with two different dry objectives, namely 4X and 10X and 0.5 NA. The excitation light was filtered by a dichroic mirror (FF720-SDi01, Semrock) and a BG39 Schott filter. We tested first the bubble with the fluorescein filling that was imaged with a 4X dry objective using a CCD camera to see the complete WGM at the equator. Figure 13 shows the TPF band around the equator and the TPF partially coupled back to the MBR wall. In this case, the MBR was filled with fluorescein and imaged with a 4X dry objective using a CCD camera to see the complete WGM at the equator.

**Figure 14:**
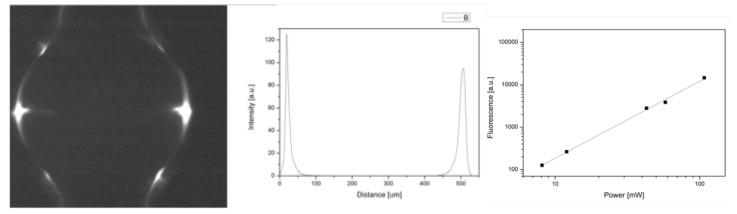
Fluorescence image of the MBR filled with $10^{-6}$ solution of Rhodamine 6G, showing the TPF lobes, the corresponding intensity plot (center) and the TPF signal versus the pump laser power in log-log scale. The red line is the linear fit with slope close to 2. The probe wavelength is $\lambda_{pump}$ = 800 nm. Reproduced with modifications from [17].

The two-photon nature of the emitted signal was validated by checking its dependence on the excitation laser power. Figure 14 shows an optical image of a MBR for an incident power of about 190 mW (the power at the focal plane is just the 30 of the incident power), its intensity plot (center); and a logarithmic representation of the TPEF signal from a Rhodamine 6G filled microbubble versus incident laser power at the focal plane. A linear fit to the data has slope close to 2, ensuring the quadratic dependence of the obtained signal. The two lobes that correspond to the WGM are clearly seen.

## 5. Conclusions

In this paper, we present an overview of the latest advances in the area of nonlinear frequency generation in WGMR. The review is limited to microspherical solid and hollow WGMR and Kerr phenomena. The Kerr effects we reviewed are third-harmonic generation, third-order sum-frequency generation, frequency combs, Kerr switching and Two-Photon Fluorescence. Stimulated Raman Scattering and Stimulated Brillouin Scattering and combination of other nonlinear phenomena such as FWM are discussed in separated subsections. WGMR are excellent platforms to understand how light, sound and matter interact. The ultimate goal of the nonlinear research in photonic devices is few or single-photon interactions to pave the way for quantum compact photonic devices at room temperature.
